# Oxytocin Facilitation of Emotional Empathy Is Associated With Increased Eye Gaze Toward the Faces of Individuals in Emotional Contexts

**DOI:** 10.3389/fnins.2020.00803

**Published:** 2020-08-11

**Authors:** Jiao Le, Juan Kou, Weihua Zhao, Meina Fu, Yingying Zhang, Benjamin Becker, Keith M. Kendrick

**Affiliations:** The Clinical Hospital of Chengdu Brain Science Institute, MOE Key Laboratory for Neuroinformation, University of Electronic Science and Technology of China, Chengdu, China

**Keywords:** autism, oxytocin, emotional empathy, eye gaze, face processing, social attention

## Abstract

One of the most robust effects of intranasal oxytocin treatment is its enhancement of emotional empathy responses across cultures to individuals displaying emotions in realistic contexts in the Multifaceted Empathy Task (MET). However, it is not established if this effect of oxytocin on emotional empathy is due to altered visual attention toward different components of the stimulus pictures or an enhanced empathic response. In the current randomized placebo-controlled within-subject experiment on 40 healthy male individuals, we both attempted a further replication of emotional empathy enhancement by intranasal oxytocin (24 IU) and used eye-tracking measures to determine if this was associated by altered visual attention toward different components of the picture stimuli (background context, human face, and body posture). Results replicated previous findings of enhanced emotional empathy in response to both negative and positive stimuli and that this was associated with an increased proportion of time viewing the faces of humans in the pictures and a corresponding decrease in that toward the rest of the body and/or background context. Overall, our findings suggest that enhanced emotional empathy following oxytocin administration is due to increased attention to the faces of others displaying emotions and away from other contextual and social cues.

**Clinical Trial Registration:** www.ClinicalTrials.gov Oxytocin Modulates Eye Gaze Behavior During Social Processing; registration ID: NCT03293511; URL: https://clinicaltrials.gov/ct2/show/NCT03293511.

## Introduction

Empathizing with others is of key importance for our social interactions. Impairments in this domain represent a major symptom in several psychiatric disorders such as autism (Lombardo et al., 2007) and depression (Tully et al., 2016; Xu et al., 2020), leading to significant problems in understanding and responding appropriately to others in social contexts. Empathy refers to a multidimensional construct including both cognitive (identifying emotions expressed by others) and emotional (being aroused by – indirect emotional empathy or feeling the same emotion – direct emotional empathy – expressed by others) facets (Shamay-Tsoory, 2011). Understanding the neurobiological regulation of these multidimensional functional domains is therefore of great importance both in the context of social neuroscience as well as to identify novel therapeutic approaches to facilitate empathy and alleviate deficits in patient populations.

In recent years, a number of studies have consistently implicated the hypothalamic neuropeptide oxytocin (OT) in the control of emotional empathy in humans (Hurlemann et al., 2010; Hubble et al., 2017a; Geng et al., 2018a, b). Intranasal OT has been demonstrated to facilitate both direct and indirect emotional empathy but not cognitive empathy as assessed by the Multifaceted Empathy Task (Dziobek et al., 2008) in male Caucasian (Hurlemann et al., 2010) as well as in both male and female Chinese subjects (Geng et al., 2018b). The latter study also found similar effects of OT on increasing emotional empathy in male subjects with both 24 and 48 IU doses. Similarly, another study using dynamic, empathy-inducing video clips reported that OT enhanced emotional but not cognitive empathy for fear (Hubble et al., 2017a), and several studies have reported that OT can increase empathy for pain experienced by others in both sexes (Shamay-Tsoory et al., 2013; Abu-Akel et al., 2015). Studies that employed concomitant functional MRI (fMRI) assessments suggest that the emotional empathy-enhancing effects of OT are neurally mediated by reduced responsiveness of the amygdala to empathy-inducing scenes (Geng et al., 2018a, b). The important role of the amygdala in emotional empathy is further supported by studies in patients with focal bilateral amygdala lesions who exhibit impaired emotional but not cognitive empathy (Hurlemann et al., 2010).

Accumulating evidence suggests that OT may generally promote face emotion recognition accuracy (Shahrestani et al., 2013), and initial studies reported increased accuracy only for difficult items in the reading the mind in the eyes test (RMET) (Domes et al., 2007), which, together, may reflect a facilitatory effect of OT on some aspects of cognitive empathy. On the other hand, more recent studies using the RMET have either only reported effects in individuals with low pretreatment empathy (Feeser et al., 2015), low social proficiency, or higher maternal love withdrawal (Riem et al., 2014), or failed to find any effects at all (Radke and de Bruijn, 2015). Thus, to date the most consistent findings indicate a facilitation of emotional empathy by intranasal OT.

There is also increasing evidence for OT playing a key role in enhancing attention toward social stimuli in a person- and context-dependent manner, and this forms the basis of the proposed “social salience hypothesis” (see Shamay-Tsoory and Abu-Akel, 2016). In support of this hypothesis, studies employing selective attention paradigms have demonstrated that OT increases attention toward social stimuli but not non-social ones (Xu et al., 2015, 2019) and promotes switching attention away from interoceptive information toward external social cues via modulating the anterior insula, a core region of the salience network (Yao et al., 2018). Studies that acquired eye-tracking indices as a behavioral measure of attentive and salience processing additionally reported that OT increases the time spent viewing social relative to non-social stimuli across a number of different paradigms (Eckstein et al., 2019; Le et al., 2020 – using the same subjects as in the current study). A number of studies have reported that OT specifically increases gaze toward the eye region of both static and dynamic facial stimuli in either healthy or autistic individuals (Guastella et al., 2008; Andari et al., 2010; Domes et al., 2013; Auyeung et al., 2015), although others have reported no effects (Domes et al., 2010; Lischke et al., 2012; Hubble et al., 2017b). Saccades from the mouth to the eye region are also increased following OT for fear expression faces and are generally associated with amygdala responses (Gamer et al., 2010). Inconsistencies between the previous studies may be explained by a number of factors such as subject sex, whether stimuli are presented statically or dynamically and whether participants were asked to passively view or actively evaluate the presented stimuli. In our own previous study in the current cohort of subjects, we found that OT primarily increased the proportion of time spent viewing the eyes relative to the nose for static fearful, but not other emotional facial expressions presented passively (Le et al., 2020). In the context of visual stimuli-evoked empathic responses, only one previous study has directly investigated the effects of intranasal OT and reported that OT increased eye gaze across dynamic expressions of sadness, happiness, pain, or fear (Hubble et al., 2017a), and empathic empathy for fearful faces. However, this study found no correlation between the proportion of gaze time toward the eyes and empathy ratings. Another study has also reported no effects of OT on gaze toward positive, negative, and neutral valence scenes depicting both humans and objects despite finding effects on neural responses to them (Lischke et al., 2017). We have therefore investigated the effects of OT on eye gaze toward different components of negative and positive valence stimuli designed to evoke empathic responses and their association with its effects on enhancing emotional empathy using the Multifaceted Empathy Task (MET).

The current preregistered pharmacological eye-tracking study employed a randomized within-subject placebo-controlled design to investigate the effects of intranasal OT (24 IU) on patterns of eye gaze in male subjects performing the MET and to replicate previous findings demonstrating OT-induced facilitation of emotional empathy (Hurlemann et al., 2010; Geng et al., 2018a, b). We only included male subjects in the current study due to the main focus of our clinical trial being relevance to autism and, additionally, because we had already established that there are no sex-dependent effects of OT on responses in the MET (Geng et al., 2018b). Based on the previous studies reporting that OT facilitated facial emotion recognition and eye gaze toward facial stimuli, we hypothesized that the OT-induced facilitation of emotional empathy would be associated with increased gaze time toward the face and away from other less socially salient features of both positive and negative valence picture stimuli. We also hypothesized that the observed effects of OT on emotional empathy ratings and time spent viewing faces would be associated with individual variations in autistic and/or empathic traits.

## Materials and Methods

### Participants

Forty healthy male adults (mean age ± SEM = 20.8 ± 0.38) were recruited via university advertisement. Only male subjects were recruited due to the main focus on potential relevance to autism and because the majority of previous studies have also only used male subjects. In a within subject design study, each participant underwent the experimental paradigm twice and with a between assessment and treatment period of approximately 2 weeks (mean ± SEM = 14.86 ± 0.16 days). Subjects were randomly assigned to receive either OXT (24 IU OXT in water, 0.9% sodium chloride, and glycerol supplied by the Sichuan Meike Pharmaceutical Company, China) or placebo (PLC containing all ingredients except for the neuropeptide and supplied by the same company) intranasal administration. The order of receiving OXT or PLC was counterbalanced across subjects. Subject number was determined by an *a priori* power analysis (using G^∗^power version 3.1.9.4 with α = 0.05 and a 0.5 correlation between repeated measures), which showed that a within-subject design would achieve >80% power for a medium effect size both with ANOVAs (*F* = 0.25, partial eta squared, 0.06) and *post hoc* pairwise comparisons (Cohen’s *d* = 0.5) with a total of 36 subjects. Finally, 40 subjects were finally recruited to compensate for potential subject dropout and data collection issues. Subjects were required to abstain from caffeine, alcohol, nicotine, or other psychoactive substances in the 24 h before each experiment, and initial interviews confirmed the self-reported absence of current or previous psychiatric illness, drug, or alcohol abuse. All participants had normal or corrected to normal vision. The study had full approval from the local ethics committee of the University of Electronic Science and Technology of China, and procedures were in accordance with the latest revision of the declaration of Helsinki. The study was preregistered at clinical trials.gov (Trial registration ID: NCT03293511; URL: https://clinicaltrials.gov/ct2/show/NCT03293511). The experiment was part of a larger study on the effects of intranasal OT on social attention during which subjects completed seven different tasks. Data from the other tasks involving social versus non-social stimuli and face emotion processing presented immediately prior to the current empathy task have been published elsewhere (Le et al., 2020). All subjects signed written informed consent and were paid for participation (100RMB).

### Experimental Procedure

In a double-blind placebo-controlled within-subject design, subjects self-administered the nasal sprays (24 IU, three puffs per nostril, each containing 4 IU of OXT or the same number of puffs with the PLC spray) following a standardized protocol (Guastella et al., 2013). At the first visit before treatment administration, participants initially completed a set of Chinese version questionnaires to evaluate mood and personality traits as a control for possible confounders due to any pretreatment group differences [State Trait Anxiety Inventory (STAI), Shek, 1993; Childhood Trauma Questionnaire (CTQ), Bernstein et al., 2003; Zhao et al., 2005]. The Positive and Negative Affect Schedule (PANAS; Watson et al., 1988) was completed three times: before treatment administration (t1) and before (t2) and after the task (t3) in order to assess possible treatment effects on mood. For the assessment of associations with experimental findings in the two groups in relation to autistic traits and empathy Chinese versions of the Autism Spectrum Quotient (AQ; Lau et al., 2013), social responsivity scale (SRS; Gau et al., 2013), and interpersonal responsivity index (IRI; Davis, 1980) were used. All subjects started the eye-tracking task 45 min after receiving the nasal spray.

The empathy task paradigm used was a Chinese version of the MET originally used in Caucasian subjects (Dziobek et al., 2008; Hurlemann et al., 2010) and described previously (Geng et al., 2018b). An adapted and shortened version of the original task was employed in the current eye-tracking study using identical real-life picture stimuli (30 positive and 30 negative valence) of people (both sexes and variable ages) displaying strong positive or negative emotions via face expressions and body posture in a particular context (displayed in the background of the picture). Stimuli were presented in a randomized order for 3 s followed by a fixation cross for 3 s (total task duration = 360 s). Subjects were required to view the stimuli passively. After the eye-tracking task, subjects were shown the picture stimuli again in a different randomized order and required to rate each one for how much they felt the same feelings as the person in the picture (i.e., direct emotional empathy) on a scale of 1–9 (1 = not at all and 9 = very strongly). Pictures were presented for a maximum of 4 s, and the rating scale was displayed under each picture. Subjects indicated their rating score by using a keyboard to move a cursor across the scale on the display screen. For flow charts showing the whole experimental procedure, see Supplementary Figure S1.

### Eye-Tracking Data

Eye-gaze data were recorded using an eye tracker (Tobii TX300, Tobii, Danderyd, Sweden), which employs infrared (IR) light-emitting diodes and IR camera to measure corneal reflections and calculate the eye-gaze direction. All gaze data were recorded at 300 Hz sampling rate with a gaze accuracy of 0.4°. Recording and stimulus presentation were conducted using Tobii Pro Studio, E-prime 2.0 software, and E-Prime Extensions for Tobii (Psychology Software Tools, Pittsburgh, PA, United States). All raw data with at least 80% gaze weight were analyzed using Tobii studio. The primary measure collected was total fixation duration, although additionally the number and duration of individual fixations were measured in order to help interpret which were contributing to observed significant changes in overall fixation duration.

### Statistical Analyses

All statistical analyses were performed using SPSS 22 (SPSS Inc., Chicago, IL, United States). Three-way repeated ANOVAs were performed with within-subject factors (treatment – OXT, PLC), AOI (background, body, and face), and valence (positive and negative) and percentage of total fixation duration as dependent variable. Separate analyses were also performed using percentage of fixation counts and individual fixation durations as dependent variables. The wide range of orientations and sizes of the faces of individuals portrayed in the picture stimuli of the MET effectively precluded a finer grained analysis of individual face features as AOIs. Similar analyses were performed with percentage of fixation counts and mean duration of individual fixations as dependent variables in order to determine whether alterations in total fixation duration were associated with altered numbers or durations of individual fixations. Significant (*p* < 0.05) interactions were explored using Bonferroni-corrected *post hoc* tests. Associations between eye-tracking variables and emotional empathy ratings and autistic and empathy traits (AQ, SRS, and IRI scores) were analyzed using Spearman correlation. To correct for multiple comparisons involving behavioral traits (three questionnaires), the threshold *p* value was adjusted to *p* < 0.0167 for the correlation analyses (e.g., *p* = 0.05/3 = 0.0167).

## Results

### Effects of Treatment on Mood and Overall Gaze Time

Independent *t* tests revealed no significant differences in terms of mood and personality traits between subjects with the two different treatment orders (see Supplementary Table S1). A two-way repeated ANOVA with two treatment × three time points for PANAS scores revealed no significant main or interaction treatment effects on positive or negative mood (all *p*’s > 0.665). There was no significant difference in the total amount of time subjects spent viewing the screen between treatments (*t* = 0.367, *p* = 0.716). Thus, OT had no overall effect on time spent viewing the stimulus screen.

### Effects of Oxytocin on Emotional Empathy and Patterns of Eye Gaze

**FIGURE 1:**
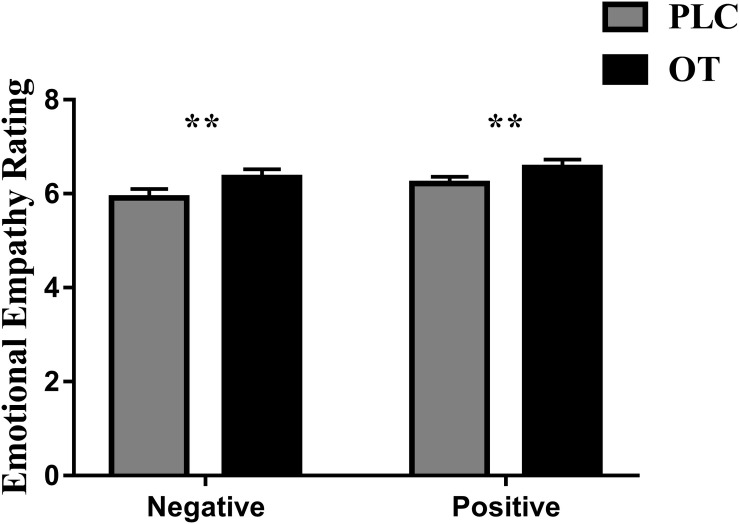
The effects of oxytocin (OT) on emotional empathy ratings. Subjects were asked to rate positive and negative valence pictures on: “how much do you feel the same as the person in the picture (i.e., direct emotional empathy)” from 1 to 9 (1 = not at all and 9 = very strongly). Error bars represent SEM. **p < 0.01, OXT vs. PLC and positive vs. negative valence.

Five subjects were excluded from analysis in this task due to technical problems with data collection on one or both of their experimental sessions. Thus, *n* = 35 subjects were included in the final analysis. We first confirmed that we could replicate previous observations that OT enhanced emotional empathy ratings. Paired *t* tests revealed that OT significantly increased empathy ratings for both negative [*t*_(1,34)_ = 2.804, *p* = 0.008, Cohen’s *d* = 0.486] and positive valence stimuli [*t*_(1,34)_ = 2.715, *p* = 0.010, Cohen’s *d* = 0.469] compared to PLC (see Figure 1). There was no treatment difference in rating scores (i.e., OT minus PLC) for positive and negative valence pictures (*p* = 0.549) indicating that OT had a similar effect on both valences.

**FIGURE 2:**
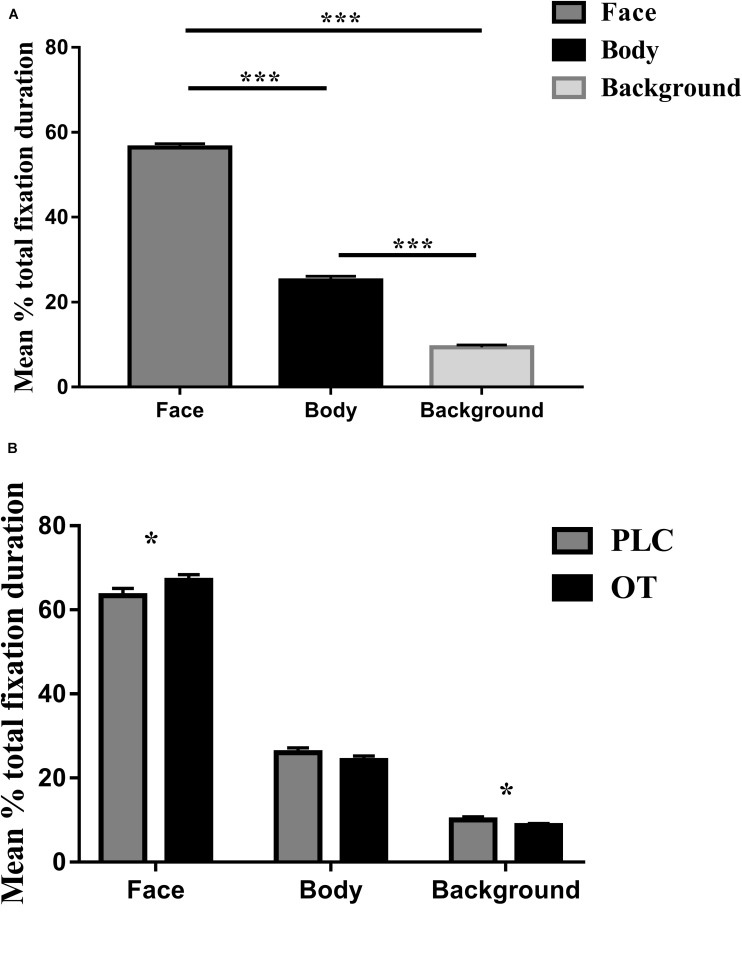
The effects of oxytocin (OT) treatment on eye gaze directed toward the face, body, or background regions during the emotional empathy (EE) task. **(A)** The mean percentage total fixation duration of face, body, and background across two valence (positive, negative). **(B)** The effects of oxytocin (OT) treatment on mean percentage total fixation duration of face, body, and background across two valence (positive, negative). Error bars represent SEM. ***p < 0.001, *p < 0.05, OXT vs. PLC.

For the eye-tracking component of the study, we used three-way ANOVAs with treatment (OT and PLC), AOIs (human face, human body, and background), and valence (positive and negative) as factors. For the primary dependent variable (proportion of time spent viewing the three AOIs relative to the whole screen), a main effect of AOI [*F*_(2,68)_ = 501.836, *p* < 0.001, partial η^2^ = 0.937] but not treatment [*F*_(1,34)_ = 1.250, *p* = 0.271] or valence [*F*_(1,34)_ = 0.142, *p* = 0.709] was observed. The main effect of AOI was due to subjects spending proportionately more time viewing human faces and bodies compared with the background (*p*’s < 0.001) and on human faces as compared to bodies (*p* < 0.001) (see Figure 2A). There were also significant treatment × AOI [*F*_(2,68)_ = 5.898, *p* = 0.013, partial η^2^ = 0.148] and AOI × valence [*F*_(2,68)_ = 21.737, *p* < 0.001, partial η^2^ = 0.390] interaction effects. *Post hoc* Bonferroni corrected *t* tests revealed that OT increased the percentage of time spent viewing faces [*t*_(1,34)_ = 2.642, *p* = 0.012, Cohen’s *d* = 0.525] but correspondingly decreased that for viewing the background [*t*_(1,34)_ = −2.291, *p* = 0.028, Cohen’s *d* = 0.559] (see Figure 2B). For the AOI × valence interaction effect *post hoc* tests also revealed that for negative compared with positive valence stimuli, subjects spent a greater proportion of time viewing the face (*p* = 0.012) and background (*p* < 0.001) but less for the body (*p* < 0.001). There were no treatment × valence [*F*_(1,34)_ = 0.482, *p* = 0.492] or treatment × regions × valence [*F*_(2,68)_ = 0.260, *p* = 0.746] interaction effects.

Identical ANOVA analyses were conducted for proportion of fixation counts and individual fixation durations. For proportion of fixation counts, we found similar results as for proportion of total viewing time, indicating that the latter were mainly due to an increased number of fixations. There was a significant main effect of AOI [*F*_(2,68)_ = 514.768, *p* < 0.001, partial η^2^ = 0.938] but not for either treatment [*F*_(1,34)_ = 1.830, *p* = 0.185] or valence [*F*_(1,34)_ = 0.000, *p* = 0.993]. The AOI main effect again reflected a higher proportion of fixation counts to the face and body compared with the background (*p*’s < 0.001) and for the face compared to the body (*p* < 0.001). There were also significant treatment × region [*F*_(2,68)_ = 4.518, *p* = 0.030, partial η^2^ = 0.117] and valence × region [*F*_(2,68)_ = 28.573, *p* < 0.001, partial η^2^ = 0.457] interaction effects. *Post hoc* Bonferroni-corrected tests revealed that OT increased the percentage of fixation counts for the face region [*t*_(1,34)_ = 2.968, *p* = 0.024, Cohen’s *d* = 0.454]. For the AOI × valence interaction, *post hoc* tests also revealed that for negative compared with positive valence stimuli, subjects showed a higher proportion of fixation counts on the face (*p* = 0.001) and background (*p* < 0.001) but less for the body (*p* < 0.001). There were no significant treatment × valence [*F*_(1,34)_ = 0.041, *p* = 0.841] or treatment × regions × valence [*F*_(2,68)_ = 2.005, *p* = 0.147] interaction effects. For individual fixation durations, there was only a significant main effect of AOI [*F*_(2,68)_ = 65.263, *p* < 0.001, partial η^2^ = 0.657] due to subjects exhibiting longer durations of individual fixations toward the face compared to body and background (all *p*’s < 0.001) and to the background compared to the body (*p* = 0.001).

### Associations Between Eye-Gaze Measures and Emotional Empathy Ratings and Autistic and Empathic Traits

**FIGURE 3:**
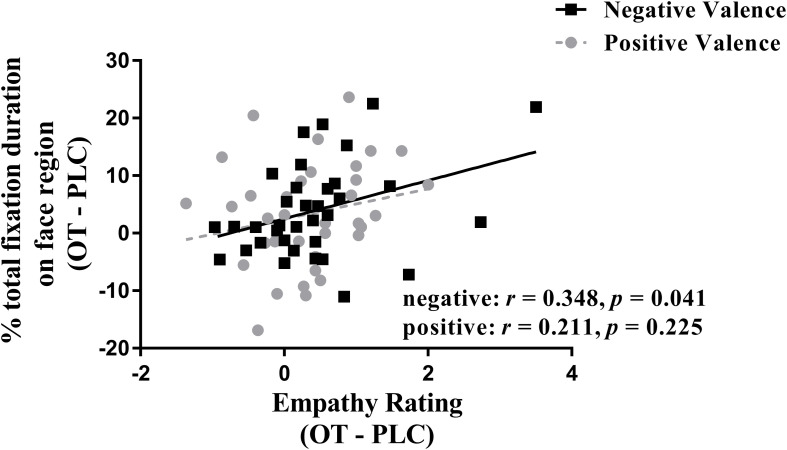
Correlation (Spearman) between emotional empathy ratings and percentage of total fixation duration using treatment differences scores (i.e., OT minus PLC treatment). Tfd, total fixation duration. Negative face: face region in negative valence pictures. Positive face: face region in positive valence pictures. Emotional empathy ratings for “how much do you feel the same as the person in the picture (i.e., direct emotional empathy)” from 1 to 9 (1 = not at all and 9 = very strongly). OT – PLC, OT minus PLC treatment condition.

We investigated correlations between treatment difference scores (i.e., OT – PLC) for both ratings and eye-tracking measures using Spearman tests. The results showed that the difference between percentage of total fixation duration for the face was positively correlated with the difference in rating scores for negative valence pictures (*r* = 0.348, *p* = 0.041), although not for positive ones (*r* = 0.211, *p* = 0.225) (see Figure 3). However, the same difference analysis for the percentage of fixation counts revealed significant correlations for both negative (*r* = 0.508, *p* = 0.002) and positive valence pictures (*r* = 0.340, *p* = 0.046) and on the body region for negative valence pictures (*r* = −0.392, *p* = 0.02) but not positive valence ones (*r* = −0.126, *p* = 0.47). Thus overall, the effects of OT on patterns of eye gaze were generally associated with those on emotional empathy ratings, although most strongly for negative valence pictures.

Spearman correlation analyses revealed no significant correlations between autistic (AQ and SRS) or empathic (IRI) traits and the percentages of total fixation duration and fixation counts on each region (all *p*’s > 0.113).

## Discussion

In the current study, we used eye tracking to investigate the effects of intranasal OT on emotional empathy and visual attention to different features of real life pictures in the MET. Results first confirmed previous findings that OT increases emotional empathy in both negative and positive emotional contexts (Hurlemann et al., 2010; Geng et al., 2018a, b). Second, results showed that OT increased both the proportion of time spent viewing the faces of the individuals portrayed in the pictures while correspondingly decreasing them to other features in the pictures. Furthermore, there was a significant positive association between the effect of OT on increasing the proportion of viewing time and/or fixation counts on the face and on increasing emotional empathy ratings, particularly for negative valence pictures. Overall, these findings provide a further replication of the finding that OT enhances emotional empathy and that this effect is associated with greater attention toward the faces of individuals displaying both negative and positive emotions and correspondingly reduced attention toward other contextual information.

Our findings that OT increased time spent viewing the faces of individuals expressing either positive or negative emotions in real-life contexts and that these were associated with parallel increased ratings of emotional empathy for negative, and to a lesser extent positive valence stimuli, support the conclusion that it may increase emotional empathy by enhancing the salience of the social stimuli. Additional support for this conclusion comes from observations in parent–offspring interactions. Mothers with higher plasma OT concentrations have been reported to pay more attention to their baby especially when the baby is distressed, implying that mothers with higher OT concentrations pay more attention to salient cues from their baby and respond more empathically toward them (Kim et al., 2014). Correspondingly, young children with higher saliva OT concentrations pay greater attention to the eye region of the faces (Nishizato et al., 2017).

There were also some indications that reductions in gaze toward other regions of the pictures were negatively associated with emotional empathy ratings. These findings contrast to some extent with a previous study using dynamic (video clip) stimuli reporting that OT increased emotional empathy for fearful faces and time spent viewing the eyes of these faces (and also happy, sad, and pain) but with an inverse relationship between them (Hubble et al., 2017a). The differences in findings may be explained by the different stimuli used, a static compared to dynamic presentation, and that in our current study, we focused on gaze toward the face as a whole rather than the eyes. We also did not focus on the specific individual emotions expressed but only whether they were in negative or positive valence situations. Another study has also reported no effects of OT on eye-gaze patterns toward positive, neutral, and negative valence pictures despite having effects on neural responses (Lischke et al., 2017). However, this latter study included both social and non-social pictures and not specifically intended to evoke empathic responses.

Subjects spent significantly more time viewing the faces and the backgrounds for negative compared with positive valence pictures, suggesting that they may pay greater attention to threatening salient cues, although pictures depicting sadness were also included. However, there was no treatment × valence interaction effect, indicating that OT had similar effects on increasing attention to salient cues for both positive and negative valence pictures. Emotional empathy ratings were also similar for positive and negative valence pictures, and altered ratings following OT were not significantly different.

We did not find any significant associations between eye-gaze parameters or emotional empathy ratings and scores on either autistic (AQ and SRS) or empathic (IRI) traits. In our previous study, we also found only marginal negative associations between AQ scores and emotional empathy ratings following PLC treatment (Geng et al., 2018a), and so a tentative conclusion from both studies is that the relationship between trait autism and emotional empathy and associated gaze toward the face in the MET is weak at best in healthy subjects. Given the greater evidence for altered patterns of eye gaze in clinical autism populations when viewing faces or other social stimuli (Chita-Tegmark, 2016; Kou et al., 2019), it is possible that they would exhibit stronger associations between symptom severity and eye-gaze and empathy ratings in the MET. Similarly, we did not find any associations with trait empathy scores as measured by the total IRI, possibly suggesting that the latter test is perhaps more sensitive for all aspects of empathic behavior rather than specifically for emotional empathy.

Several limitations should be acknowledged in the current study. First, we only included male subjects, and there is increasing evidence for sex-dependent effects of OT (Gao et al., 2016; Luo et al., 2017; Lieberz et al., 2019), although we have previously shown no sex-dependent effects of OT on emotional empathy or amygdala responses in the MET (Geng et al., 2018b). Two other studies showing effects of OT on pain empathy have also not found sex-dependent effects (Shamay-Tsoory et al., 2013; Abu-Akel et al., 2015), and so we would predict that similar effects of OT on patterns of eye gaze during the MET would occur in female subjects. Second, we could only investigate effects of OT on gaze toward the whole face region due to the nature of the MET pictures and so were unable to investigate if the eye region was of most importance. Finally, the picture stimuli presented were static, and it is possible that results using dynamic pictures could be different.

In summary, our findings in the current study demonstrate that the effects of OT in increasing emotional empathy responses to positive and negative expressions of emotions in real-life contexts are associated with an increase in time viewing the face region of protagonists in the pictures and correspondingly less to other salient features. This provides further support for an important general role of OT in shifting attention toward the most salient features of social stimuli in line with the social salience hypothesis (Shamay-Tsoory and Abu-Akel, 2016) and may contribute to greater attention and empathic responses in more specific contexts such as parent–offspring interactions.

## Data Availability Statement

The raw data supporting the conclusions of this article will be made available by the authors, without undue reservation.

## Ethics Statement

The studies involving human participants were reviewed and approved by Ethics Committee of the University of Electronic Science and Technology of China. The patients/participants provided their written informed consent to participate in this study.

## Author Contributions

JL, JK, and KK designed the experiment. JL, MF, and YZ conducted the experiment. JL and WZ analyzed the data. JL drafted the manuscript. BB and KK interpreted the results, revised it critically for important intellectual content, and finalized the manuscript for submission. All authors contributed to manuscript revision and read and approved the submitted version.

## Conflict of Interest

The authors declare that the research was conducted in the absence of any commercial or financial relationships that could be construed as a potential conflict of interest.
