# Supplementary material for: Oxytocin Facilitation of Emotional Empathy Is Associated With Increased Eye Gaze Toward the Faces of Individuals in Emotional Contexts
Source: Front Neurosci. 2020 Aug 11;14:803. doi: 10.3389/fnins.2020.00803 (PMC7432151; doi:10.3389/fnins.2020.00803)
Supplement: Supplementary file 1 [file Data_Sheet_1.docx]

Supplementary Material

**Table S1** Participant demographic and questionnaire data in individuals receiving OT or PLC treatment first (means and SEMs).

| Measurements | First taking OT  (n = 20) | First taking PLC  (n = 20) | t | P |
| --- | --- | --- | --- | --- |
| Age | 21(0.60) | 21(0.48) | -0.394 | 0.696 |
| SRS | 59(6.79) | 51(4.31) | 0.957 | 0.344 |
| AQ | 20(1.12) | 21(1.36) | -0.823 | 0.416 |
| SAI | 36(2.02) | 37(2.00) | -0.228 | 0.821 |
| TAI | 41(1.85) | 41(1.77) | 0.313 | 0.756 |
| CTQ | 27(1.24) | 27(1.17) | -0.147 | 0.884 |
| IRI | 48(1.50) | 48(1.83) | 0.169 | 0.867 |

Group differences were analyzed by t test. SRS: Social Responsiveness Scale; AQ: Autism-Spectrum Quotient; SAI, TAI: State and Trait Anxiety Inventory; CTQ: Childhood Trauma Questionnaire; IRI: Interpersonal responsivity index.


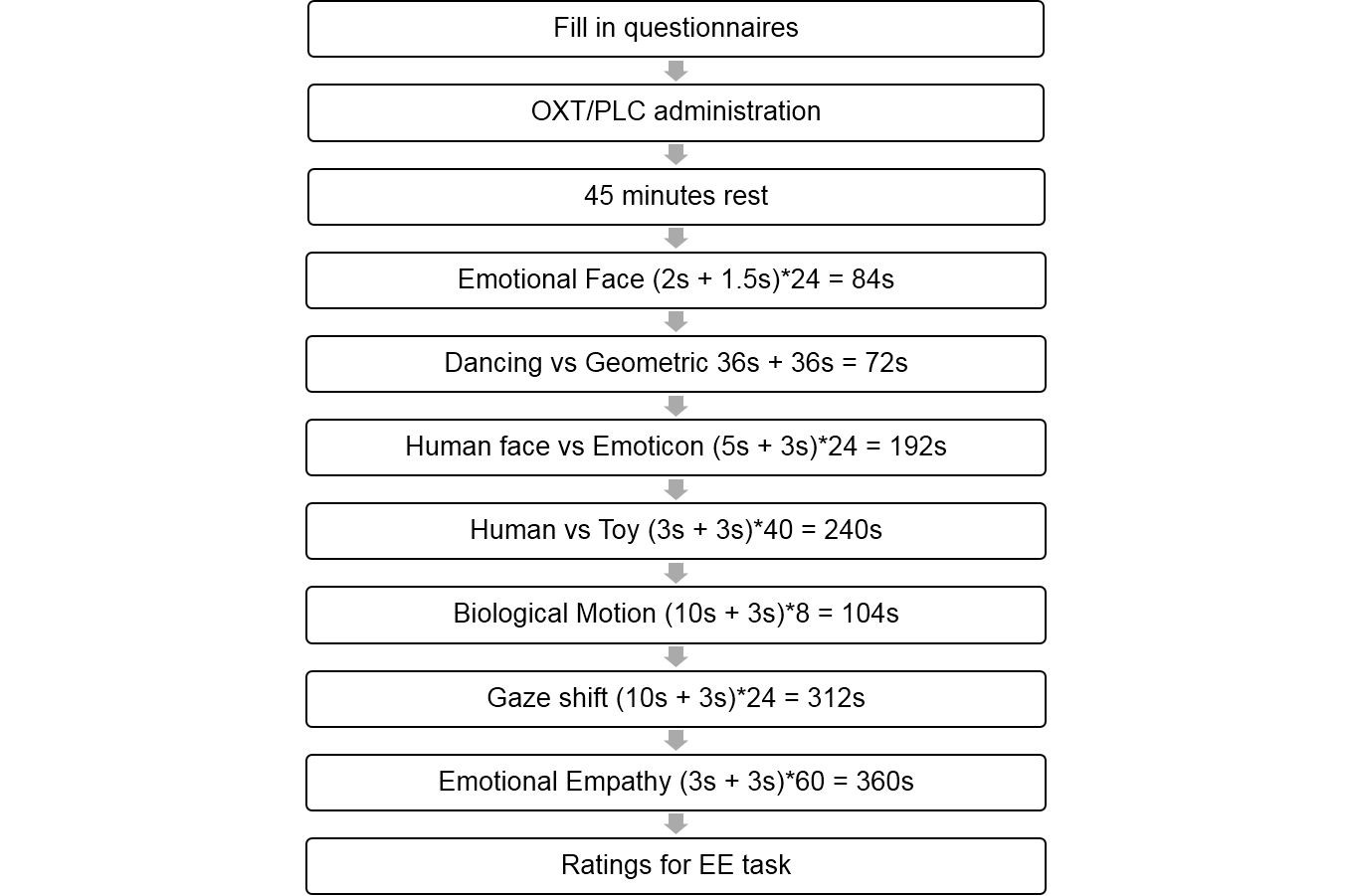


**Figure S1.** Flow chart of experiment with the fixed order of the eye tracking task. S (seconds); EE (emotional empathy).
