# Supplementary material for: Oxytocin Facilitation of Emotional Empathy Is Associated With Increased Eye Gaze Toward the Faces of Individuals in Emotional Contexts
Source: Front Neurosci. 2020 Aug 11;14:803. doi: 10.3389/fnins.2020.00803 (PMC7432151; doi:10.3389/fnins.2020.00803)
Supplement: Supplementary file 3 [file Table_2.doc]

**
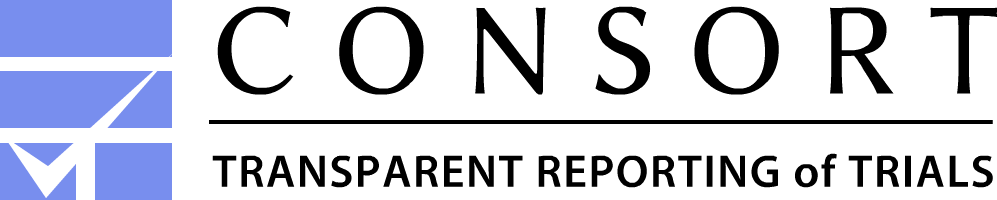
**

**CONSORT 2010 Flow Diagram**

Oxytocin facilitation of emotional empathy is associated with increased eye-gaze towards the faces of individuals expressing emotion

Jiao Le, Juan Kou, Weihua Zhao, Meina Fu, Yingying Zhang, Benjamin Becker, Keith M Kendrick

**Allocation**

**Analysis**

**Follow-Up**

**Enrollment**

Assessed for eligibility (n = 40)

Excluded (n = 0)

♦  Not meeting inclusion criteria (n = 0)

♦  Declined to participate (n = 0)

♦  Other reasons (n = 0)

Analysed (n = 16)

Excluded from task analysis (technique problem) (n = 2)

Excluded from task analysis (failed with presentation) (n =2)

Lost to follow-up (n = 0)

Discontinued intervention (n = 0)

Allocated to intervention (oxytocin first group) (n = 20)

♦ Received allocated intervention (n = 20)

♦ Did not receive allocated intervention (n = 0)

Lost to follow-up (n = 0)

Discontinued intervention (n = 0)

Allocated to intervention (Placebo first group) (n = 20)

♦ Received allocated intervention (n = 20)

♦ Did not receive allocated intervention (n = 0)

Analysed (n = 19)

Excluded from task 2, 3, 4, 5 analysis (technique problem) (n = 1)

Randomized (n = 40)
